# A WS_2_-gold nanoparticle heterostructure-based novel SERS platform for the rapid identification of antibiotic-resistant pathogens

**DOI:** 10.1039/d0na00141d

**Published:** 2020-03-31

**Authors:** Avijit Pramanik, Dalephine Davis, Shamily Patibandla, Salma Begum, Priyadarshini Ray, Kaelin Gates, Ye Gao, Paresh Chandra Ray

**Affiliations:** Department of Chemistry and Biochemistry, Jackson State University Jackson MS USA paresh.c.ray@jsums.edu +1 6019793674

## Abstract

The emergence of antibiotic-resistant bacteria is the biggest threat to our society. The rapid discovery of drug resistant bacteria is very urgently needed to guide antibiotic treatment development. The current manuscript reports the design of a 2D–0D heterostructure-based surface enhanced Raman spectroscopy (SERS) platform, which has the capability for the rapid identification of the multidrug resistant strain of *Salmonella DT104*. Details of the synthesis and characterization of the heterostructure SERS platform using a two dimensional (2D) WS_2_ transition metal dichalcogenide (TMD) and zero dimensional (0D) plasmonic gold nanoparticles (GNPs) have been reported. The current manuscript reveals that the 2D–0D heterostructure-based SERS platform exhibits extremely high Raman enhancement capabilities. Using Rh-6G and 4-ATP probe molecules, we determined that the SERS sensitivity is in the range of ∼10^−10^ to 10^−11^ M, several orders of magnitude higher than 2D-TMD on its own (10^−3^ M) or 0D-GNPs on their own (∼10^−6^ to 10^−7^ M). Experimental and theoretical finite-difference time-domain (FDTD) simulation data indicate that the synergistic effect of an electromagnetic mechanism (EM) and a chemical mechanism (CM) on the heterostructure is responsible for the excellent SERS enhancement observed. Notably, the experimental data reported here show that the heterostructure-based SERS has the ability to separate a multidrug resistance strain from a normal strain of *Salmonella* by monitoring the antibiotic–pathogen interaction within 90 minutes, even at a concentration of 100 CFU mL^−1^.

## Abstract 2

The emergence of antibiotic-resistant bacteria is the biggest threat to our society.

## Graphical abstract

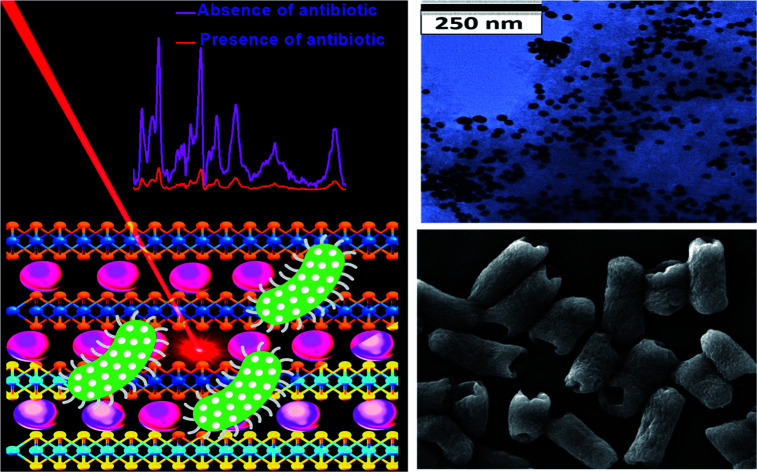

## Introduction

After the discovery of graphene, two-dimensional (2D) materials have drawn significant attention in the scientific community due to their excellent structural, physical, optical and electronic properties.^1–5^ In recent years, heterostructural building blocks using 2D, one dimensional (1D) and zero dimensional (0D) materials have opened up unique opportunities for fundamental scientific studies due to the combined advantages of the individual materials.^6–12^ Recent reports demonstrated that heterostructures exhibit exceptional optical and electronic properties that are not available in the individual material.^4–10^

Due to its high sensitivity, nondestructive character and fingerprint identification capability, SERS is highly promising in biomedical technology.^13–20^ In the last two decades we and others have reported that extremely weak Raman signals can be enhanced significantly using a proper design of the SERS substrate *via* the electromagnetic mechanism (EM) and chemical mechanism (CM).^16–24^ Zero dimensional plasmonic nanoparticles such as gold and silver nanoparticles have been used extensively as SERS materials, where Raman intensity can be enhanced by ∼10^6^*via* EM & CM.^13–20^ On the other hand, in the last few years SERS substrates based on two-dimensional (2D) transition-metal dichalcogenides (TMD) have been reported, where Raman intensity can be enhanced by ∼10^2^*via* CM as well as due to the resonance with near- and far-field optical properties.^11,16,20–24^ To take the advantages of the EM and CM effect from both types of materials, in the current manuscript we have reported the development of novel heterostructure material-based SERS using 2D WS_2_ TMD and 0D plasmonic gold nanoparticles (GNPs). The reported data show a remarkably high SERS sensitivity of ∼10^−10^ to 10^−11^ M from the heterostructure.

Antibiotic resistant bacteria, which are a nightmare for medical treatment, are responsible for more than a million deaths in the world every year.^25–30^ As per the Center for Disease Control and Prevention (CDC), antibiotic resistance is one of the biggest public health challenges in the 21^st^ century.^26^ As a result, there is a huge urgency in finding rapid and cost-effective assays which will have the capability for rapid and accurate determination of the appropriate antibiotic for an infection in a timely manner.^31–42^ Driven by this need, the current manuscript demonstrates that 0D–2D heterostructure-based SERS is capable of rapid identification of drug resistant bacteria by monitoring structural changes in the pathogen's cell wall during antibiotic treatment. Reported data show that the SERS can be used for the identification of multidrug resistant *Salmonella DT104* and a normal strain *Salmonella Typhi* antimicrobial susceptibility test using Augmentin antibiotics, even at the concentration of 100 CFU mL^−1^ experimental data reported here indicates that heterostructure-based SERS has the capability of antimicrobial susceptibility testing in less than 2 hours, whereas the “gold standard” broth microdilution (BMD) test takes 16 to 20 h in clinics until the number of bacteria reaches above 10^5^ CFU mL^−1^.

## Experimental section

### Development of the heterostructure-based SERS platform

**Scheme 1:**
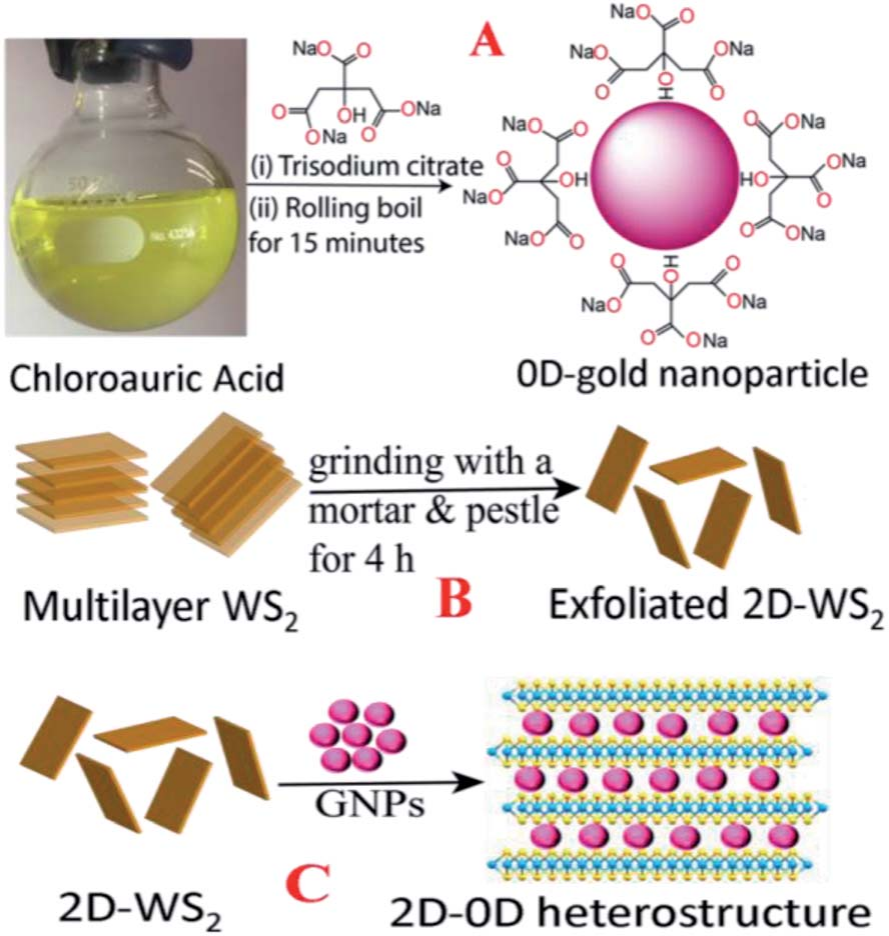
(A) A schematic diagram showing the synthetic procedure we used for the development of 0D plasmonic gold nanoparticles. (B) A schematic diagram showing the synthetic procedure we used to develop 2D-WS_2_ nanosheets. (C) A schematic diagram showing the synthetic procedure we used to develop the 0D–2D heterostructure.

**Fig. 1:**
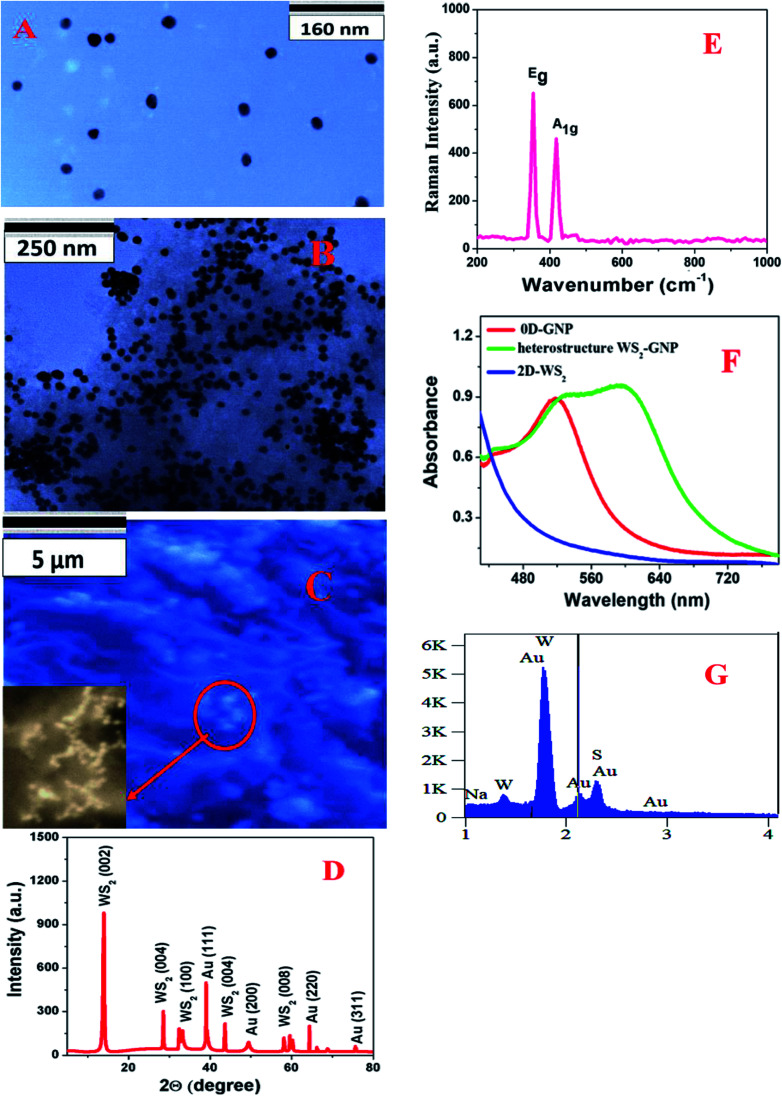
(A) A TEM image showing the morphology of the 0D-GNPs. (B) A TEM image showing the morphology of the heterostructure based on 2D-WS_2_ and 0D-GNPs, which indicates that the 0D-GNPs aggregate on 2D-WS_2_ (C) a SEM image showing the morphology of the heterostructure based on 2D-WS_2_ and 0D-GNPs. The inserted high resolution SEM image indicates that the 0D-GNPs aggregates on 2D-WS_2_. (D) The XRD spectrum from the heterostructure. (E) The Raman spectrum from the heterostructure. (F) Extinction spectra from the heterostructure, GNPs and WS_2_. (G) The EDX spectrum from the heterostructure.

0D-plasmonic- GNPs were synthesized according to the previous work by our group^18–20,24,29^ using HAuCl_4_ and trisodium citrate dihydrate, as shown in Scheme 1A. For this purpose, 1.25 mL of 10 mM HAuCl_4_ solution was completely dissolved in 50 mL of deionized water. The mixture was stirred on a hot plate and brought to a rolling boil. Next, we added 2 mL of 1% trisodium citrate dihydrate to the boiling solution. After a few minutes, a colloidal solution was formed and then we continued heating for another few minutes. After that the colloidal gold nanoparticles were centrifuged at 5000 rpm for a few hours and the supernatant was removed by decantation. The purified AuNPs were kept at 4 °C for future use. After purification, the AuNPs were characterized by electron microscopy and absorption techniques. Fig. 1A shows the transmission electron microscopy (TEM) image which indicates that the size of the 0D-GNPs is ∼25 ± 5 nm. Fig. 1F shows the absorption spectra of 0D-GNPs, which clearly show a strong plasmon band at 520 nm. The observed strong plasmon band is mainly due to the localized surface plasmonic resonance (LSPR) of the GNPs induced by the incident light.^13–18^

2D-WS_2_ nanosheets were synthesized using a simple ultrasonication route from WS_2_ powder.^16,20–22^ Briefly, commercial WS_2_ bulk powder was pelletised by a grinding miller for about 4 h, as shown in Scheme 1B. In the next step, WS_2_ nanosheets were exfoliated through liquid exfoliation followed by intercalation. For this purpose, WS_2_ nanosheets were added to H_2_SO_4_ (98%) at 90 °C for 24 h.

After that WS_2_ nanosheets were collected after centrifugation at 5000 rpm for 1 h and kept in the fridge for future use. At the end, 2D-WS_2_ nanosheets were characterized by electron microscopy and absorption techniques. Fig. 1F shows the absorption spectra of 2D-WS_2_, which shows some absorption at 400–650 nm and it is mainly due to the A1 and B1 excitonic transitions of WS_2_.^16,20–22^ The observed exitonic transition originates from the energy split of the valence-band and spin-orbital coupling, as reported before.^16,20–22^

In the next step we synthesized 0D–2D heterostructures using 2D-WS_2_ and 0D-GNPs. For this purpose, GNP solution was dropped in freshly prepared WS_2_ solutions in phosphate-buffered saline. After that the 2D-WS_2_ and 0D-GNP mixture was sonicated for 2 h at room temperature. In the next step the 2D-WS_2_ and 0D-GNP mixture was continuously stirred at very low speed overnight at room temperature for the completion of the reaction. After that the product was washed with methanol three times by centrifugation at 5000 rpm for 15 min followed by decantation. Finally, the heterostructure materials were dried under vacuum at room temperature for a week. After that the heterostructure materials were characterized by TEM, SEM, absorption, energy dispersive X-ray (EDX), X-ray diffraction (XRD) and Raman spectroscopy techniques, as reported in Fig. 1C–G. Fig. 1B shows the TEM image and Fig. 1C shows the SEM image of the freshly prepared heterostructure which indicates that 0D-GNPs are on the surface of 2D-WS_2_. Both the TEM and inserted high-resolution SEM images indicate the formation of GNP aggregates on the 2D-WS_2_ surface. EDX data from the heterostructure has been reported as Fig. 1G, which clearly shows the presence of W, S, and Au. Fig. 1D shows the powder XRD data from the heterostructure, which shows the presence of (002), (004), (006) and (008) reflections for WS_2_, and (111), (200), (220) and (3111) reflections for GNP.^17,20–22,24^Fig. 1F shows the absorption spectra of the heterostructures which clearly indicate a broad plasmon band which is due to the aggregation of 0D-GNP on the surface of 2D-WS_2_ as shown in the TEM images reported in Fig. 1B. Both the absorption spectra and TEM/SEM images clearly indicate the formation of 0D-GNP aggregates on the surface of 2D-WS2 in the heterostructure. Fig. 1E shows the Raman spectra from the heterostructure, which indicate the presence of an in-plane (E_2g_) Raman band at ∼356 cm^−1^ and an out-of-plane (A_1g_) Raman band at ∼417 cm^−1^, which is due to the W–S vibration of WS_2_.^17,20–22^

### SERS using 2D-WS2, 0D-GNPs and the heterostructure substrate

For the SERS experiment we used 1 mg mL^−1^ of the as-synthesized ‘2D-WS2’ and ‘2D–0D heterostructure’ samples. For the Raman experiments, we used a confocal Raman system with a laser excitation of 670 nm at ∼2 mW power. We used 100× magnification, and a numerical aperture of 0.9 for our experiment. We collected signals in a backscattering configuration. In our experiment, for the determination of the SERS enhancement factor on the 2D-WS2, 0D-GNP and heterostructure surfaces, we assumed that the analytes were distributed uniformly on the surface. For the Raman intensity measurements, the heterostructure platform was immersed in different concentrations of the analyte for two hours and the SERS substrate was dried under N_2_ flow. For the Raman data collection from the 2D-WS2, 0D-GNPs and heterostructure substrates with 4-ATP we used a 10 second acquisition time and 5-scan averaging, so that we could achieve a very good signal-to-noise ratio. For the Raman measurements from the bacteria, at first, we located the bacteria position using a microscope attached to a confocal Raman microscopy system, where a single bacterium or clusters of bacteria could be easily identified. After that the laser beam was focused on the bacteria through the microscope object, high quality Raman spectra from 10 to 15 spots were measured and after that the spectra were processed with a baseline removal program. In the next step, we performed averaging from the different spots, so that we could achieve a very good signal-to-noise ratio.

### FDTD simulations for full-field electromagnetic wave calculations

We used the finite-difference time-domain (FDTD) simulation for full-field electromagnetic wave calculations, as previously reported by our group.^13,18,19,24^ In this process we used numerical calculations which can be used to understand plasmon-coupling in a “hot spot”, which is responsible for the huge EM enhancement mechanism in SERS.^13–20,24^ In our calculations the electric field intensities were simulated by using a diameter of the 0D-GNPs of 25 nm which are decorated on the 2D-WS_2_ nanosheets as we observed experimentally. To keep the experimental conditions, we used an incident laser wavelength of 670 nm for our calculations. For the simulation calculations the entire process was performed with a 0.001 nm mesh resolution and 4000 fs time, as we have reported before.^13,18,19,24^

### Bacteria sample preparation

The multidrug resistant strain *Salmonella DT104*, the normal strain of *Salmonella Typhi* and growth media were purchased from the American Type Culture Collection (ATCC, Rockville, MD). We cultured these bacteria according to the ATCC protocol, as we have reported before.^18,19,24,29,30^ Once the different bacteria had grown to 10^6^ CFU mL^−1^, we used them for the experiment.

### SERS based determination of antimicrobial susceptibility

For the determination of antimicrobial susceptibility, *Salmonella Typhi* and *Salmonella DT104* bacteria were incubated with the Augmentin antibiotic for 2 hours. During this process, we measured time dependent SERS spectra of the bacteria on the heterostructure surface for two hours of incubation.

### Finding the percentage of live bacteria after exposure to the Augmentin antibiotic

We used a colony-countable plate to determine the percentage of live bacteria before and after treatment. For this purpose, we transferred antimicrobial susceptible, *Salmonella Typhi* and *Salmonella DT104* bacteria to colony-countable plates. We incubated them for 24 h at 37 °C and then the colony number was counted with a colony counter (Bantex, Model 920 A). We also used molecular probes' LIVE/DEAD® BacLightTM Bacterial Viability Kits (from Fisher Scientific) to determine the amount of antimicrobial susceptible, *Salmonella Typhi* and *Salmonella DT104* bacteria that was dead after antibiotic treatment. This kit is well known to provide a two-color fluorescence assay, which utilizes mixtures of our SYTO® 9 green-fluorescent nucleic acid stain which can bind with live bacteria and the red-fluorescent nucleic acid stain, propidium iodide, which is known to bind with dead bacteria.

### ATP leakage experiment

To better understand cell wall and membrane damage during antibiotic treatment, we performed a bacterial ATP leakage experiment using the molecular probes® ATP determination kit (Thermo Fisher Scientific). For this purpose, bacteria cultures without antibiotics were used as a reference. This ATP determination kit is based on the luminescence results from the firefly luciferase enzyme.

## Results and discussion

### Finding the Raman enhancement, stability and reproducibility

**Fig. 2:**
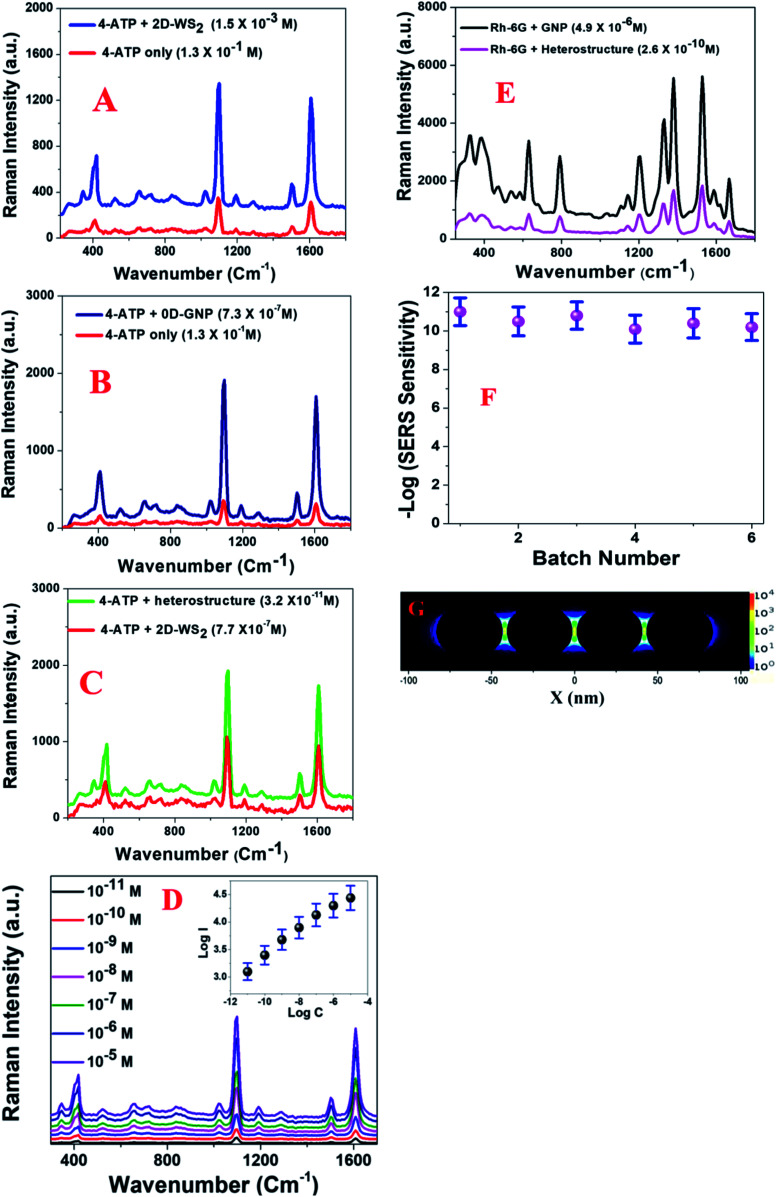
(A) Plots showing how the Raman profile from 4-ATP varies in the presence of the 2D-WS_2_ surface and without any surface. To remove the E_g_ and A_1g_ band contributions from WS_2_, we subtracted the Raman spectra of 4-ATP with the heterostructure SERS substrate from the spectrum of the heterostructure SERS substrate on its own. (B) Plots showing how the Raman profile from 4-ATP varies in the presence of the 0D-GNP surface and without any surface. (C) Plots showing how the Raman profile from 4-ATP varies in the presence of a heterostructure and GNP surface. (D) Plots showing how the Raman profile from 4-ATP varies with concentration in the presence of the heterostructure. The inserted plot shows how log(intensity at 1078 cm^−1^) varies with log(concentration of 4-ATP). (E) Plots showing how the Raman profile from Rh-6G varies in the presence of the heterostructure and the GNP surface. (F) A plot showing how log(Raman sensitivity) for 4-ATP varies between heterostructures made in different batches. (G) FDTD simulation data showing the electric field enhancement square (|*E*|^2^) profiles for a 0D-GNP assembly containing four nanoparticles.

Since the enhancement factor is the most important parameter for designing a novel Raman substrate, we determined the Raman EF using 4-aminothiophenol (4-ATP) for the 2D-WS2, 0D-GNPs and heterostructure materials. To remove the E_g_ and A_1g_ band contribution from WS_2_, we subtracted the Raman spectra of 4-ATP with the heterostructure SERS substrate from the spectrum of the heterostructure SERS substrate on its own. To find the enhancement factor for each type of material, we used the confocal Raman system with a laser excitation of 670 nm. Fig. 2A–C show the Raman spectra of 4-aminothiophenol on 2D-WS2, 0D-GNPs and the heterostructure. The reported Raman data in Fig. 2A show that significant changes in the intensities of the 4-ATP Raman peaks are observed on the 2D-WS_2_ substrate in comparison to the Raman spectrum of 4-ATP on its own. The observed Raman peaks are dominated by a_1_ vibrational modes and these are *ν*(C–C + NH_2_ bend) at ∼1590 cm^−1^ and *ν*[*ν*(C–C) + *δ*(C–H)] at ∼1078 cm^−1^.^13–20,24^ In the reported spectra, we also observed Raman peaks due to b_2_ modes at ∼1435 cm^−1^, which can be ascribed to the CC stretch in the Ph ring + NH_2_ rock.^13–20,24^ Another Raman peak due to b_2_ modes is observed at ∼1170 cm^−1^ which can be ascribed to the CH bend vibration.^13–20,24^ Similarly, we also observed a Raman band at 464 cm^−1^ due to the *ν*(C–N) + *ν*(C–S) + *γ*(CCC) vibrations.^13–20,24^

As shown in the TEM and SEM images reported in Fig. 1B and C, the distribution of GNP is not uniform. As a result, the Raman intensity may vary at different spots of the heterostructure surface due to the possible aggregation of the 0D-GNPs on the 2D surface. Our experimental results indicate that Raman intensity can vary more than 10% from one spot to another spot due to the variation of GNP aggregate sizes at different spots. As a result, we measured the SERS intensity at 10 different spots and the average values are reported in this manuscript. Using the 4-ATP probe molecule, we determined that the SERS sensitivity is in the range of ∼10^−3^ M for 2D-WS_2_. The observed Raman sensitivity from 2D-WS_2_ can be attributed to the chemical enhancement mechanism as well as to the resonance with near- and far-field optical properties.^11,16,20–24^Fig. 2B shows the Raman enhancement of 4-ATP on the 0D-GNP surface. Using the reported data in Fig. 2B, we estimated that the average SERS sensitivity is in the range of ∼10^−7^ M for 0D-GNP. The observed high SERS sensitivity for 0D-GNP can be attributed due to the strong electromagnetism as well as the chemical enhancement effect caused by the charge-transfer process happening on the ATP-GNP surface.^12–19^ Similarly, using the reported data in Fig. 2C, we estimated that the average SERS sensitivity is in the range of ∼10^−11^ M for the heterostructure and the average SERS sensitivity is in the range of ∼10^−7^ M for 0D-GNP. The observed 10^4^ higher Raman sensitivity from the heterostructure surface is due to the strong electromagnetism as well as the strong chemical enhancement capability for the heterostructure. The 4-ATP concentration dependent SERS spectra are reported in Fig. 2D, which also indicates that the SERS sensitivity is in the range of ∼10^−11^ M for the heterostructure.

Fig. 2E shows the SERS spectra from Rh-6G on 0D-GNPs and the heterostructure surface. Using the Rh-6G probe molecule, we determined that the SERS sensitivity is in the range of ∼10^−10^ M, for the heterostructure and ∼10^−6^ M for 0D-GNPs. The observed 10^4^ higher Raman sensitivity from the heterostructure surface is due to the strong electromagnetism as well as the strong chemical enhancement capability of the heterostructure. We performed the finite-difference time-domain (FDTD) simulation^13,18,19,24^ to better understand the near-field distribution around the 0D-GNPs. The calculation details have been reported before by our group.^13,18,19,24^Fig. 2G shows that the square of the field enhancement (|*E*|^2^) for the 0D-GNP “hot spots” can be around two orders of magnitude higher than that of an individual nanoparticle. It is now well documented^13–19,24^ that the Raman EF *α*|*E*|^4^. As a result, we expect to increase the Raman enhancement to around 4 orders of magnitude for the heterostructure, which is only due to the formation of aggregates by 0D-GNPs on the surface of 2D-WS_2_. To understand the reproducibility, we developed a heterostructure in five different batches using 0D-GNP and 2D-WS_2_. After that, we monitored the average Raman sensitivity using 4-ATP in 10 different spots for each batch. As reported in Fig. 2F, our experimental data show that the relative standard deviation is around ∼10%.

### Use of heterostructure-based SERS for the label-free determination of antimicrobial susceptibility

**Fig. 3:**
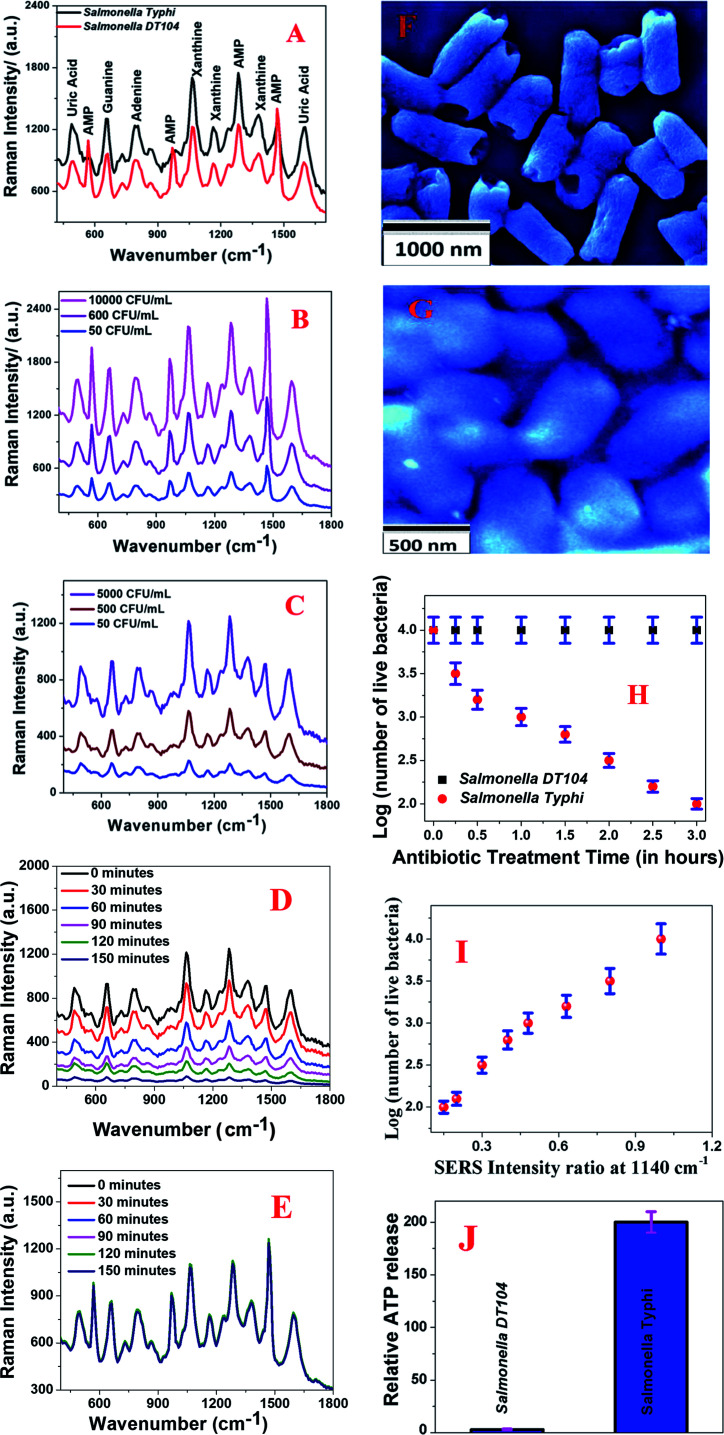
(A) The Raman spectra from the multidrug resistant strain of *Salmonella DT104* and the normal strain of *Salmonella Typhi* on the heterostructure surface. To remove the E_g_ and A_1g_ band contributions from WS_2_, we subtracted the Raman spectra of the pathogens with the heterostructure SERS substrate from the spectrum for the heterostructure SERS substrate on its own. (B) Plots showing how the Raman spectrum from the multidrug resistant strain of *Salmonella DT104* varies with concentration (CFU mL^−1^). (C) Plots showing how the Raman spectrum from the normal strain of *Salmonella Typhi* varies with concentration (CFU mL^−1^). (D) Plots showing how the time dependent Raman spectra from the normal strain of *Salmonella Typhi* vary during Augmentin antibiotic treatment. We used 1000 CFU mL^−1^*Salmonella Typhi* bacteria for this experiment. (E) A plot showing how the time dependent Raman spectra from the multidrug resistant *Salmonella DT104* vary during Augmentin antibiotic treatment We used 1000 CFU mL^−1^*Salmonella DT104* bacteria for this experiment. (F) A SEM image showing that the bacterial cell wall gets damaged when the normal strain of *Salmonella Typhi* was treated with the Augmentin antibiotic. (G) A SEM image of the *Salmonella DT104* bacteria after antibiotic treatment. It shows that the bacterial cell wall remains intact when multidrug resistant *Salmonella DT104* was treated with the Augmentin antibiotic. (H) Plots indicating the percentages of live bacteria for *Salmonella DT104* and *Salmonella Typhi* during treatment with the Augmentin antibiotic. We used 1000 CFU mL^−1^ bacteria for this experiment. (I) A plot showing how the SERS intensity at 1140 cm^−1^ decreases as the number of live bacteria decreases during treatment with the Augmentin antibiotic. We reported the ratio of SERS intensity at 1140 cm^−1^ before and after treatment. We used 1000 CFU mL^−1^*Salmonella Typhi* bacteria for this experiment. (J) Plots showing the relative cellular ATP leakage from *Salmonella DT104* and *Salmonella Typhi* after treating with the Augmentin antibiotic.

Next, to understand whether 0D–2D heterostructure-based SERS can be used for rapid and accurate determination of antimicrobial susceptibility, we used multidrug resistant-*Salmonella DT104* and a normal strain of *Salmonella Typhi*. In our approach, we used 0D–2D heterostructure-based SERS for monitoring structural changes in the pathogen's cell wall during the Augmentin antibiotic treatment. To remove the E_g_ and A_1g_ band contribution from WS_2_, we subtracted the Raman spectra of pathogens with the heterostructure SERS substrate from the spectrum for the heterostructure SERS substrate on its own. As reported in Fig. 3A–E, we observed very strong Raman peaks from the multidrug resistant *Salmonella DT104* and the normal strain of *Salmonella Typhi* in the presence of the 0D–2D heterostructure. As shown in Fig. 3A, the observed vibrational Raman bands from multidrug resistant *Salmonella DT104* and the normal strain of *Salmonella Typhi* can be assigned to the spectral contributions of different purines such as adenine, xanthine, guanine, uric acid, and adenosine monophosphate (AMP) as reported by Premasiri and Ziegler *et al.*^41,42^ By comparing the SERS data reported in Fig. 3A, we can conclude that most of the Raman peaks are very similar for multidrug resistant *Salmonella DT104* and the normal strain of *Salmonella Typhi*, except the AMP Raman bands at ∼570 cm^−1^, ∼968 cm^−1^ and ∼1386 cm^−1^, which are much stronger and unique for multidrug resistant *Salmonella DT104*. As shown in Fig. 3B and C, concentration dependent SERS data clearly indicate that the heterostructure based SERS platform can be used for the identification of multidrug resistant *Salmonella DT104* and the normal strain of *Salmonella Typhi* even at a concentration of 50 CFU mL^−1^.

Our SERS based determination of antimicrobial susceptibility is based on the fact that the Augmentin antibiotic will alter the biochemical components of the normal strain of *Salmonella Typhi* bacterial cell wall and it will induce variation in the SERS spectra. On the other hand, since multidrug resistant *Salmonella DT104* is resistant to the Augmentin antibiotic, it will not able to alter the biochemical components of the *Salmonella DT104* cell wall and as a result, there will be no variation in the SERS spectra. For the determination of antimicrobial susceptibility, *Salmonella Typhi* and *Salmonella DT104* bacteria cultures without an antibiotic were used as a reference. During the incubation of *Salmonella Typhi* and *Salmonella DT104* bacteria cultures with the Augmentin antibiotic, we measured the time dependent SERS spectra of bacteria on the heterostructure surface for two hours of incubation.

We used a bacterial viability assay kit to determine the amount of dead bacteria after antibiotic treatment. Fig. 3D shows how the characteristic Raman peaks from the *Salmonella Typhi* bacteria vary during Augmentin antibiotic exposure. We used 1000 CFU mL^−1^ of *Salmonella Typhi* bacteria for this experiment. The reported data clearly show that the Raman bands from the *Salmonella Typhi* bacteria decreased significantly during antibacterial treatment, which indicates that the observed Raman modes originated from the bacterial cell wall. To understand better, we also performed the SEM image experiment, as reported in Fig. 3F. Reported SEM data clearly shows that the bacterial cell walls are damaged during Augmentin antibiotic treatment for the *Salmonella Typhi* bacteria. On the other hand, Fig. 3E shows how the characteristic Raman peaks from the multidrug resistant *Salmonella DT104* bacteria varies during Augmentin antibiotic exposure. We used 1000 CFU mL^−1^ of *Salmonella DT104* bacteria for this experiment.

The reported data clearly shows that the Raman bands remain unaltered during the antibacterial treatment, which indicates that there may be no damage to the bacterial cell wall during antibiotic treatment for *Salmonella DT104*. Fig. 3G shows the SEM image of *Salmonella DT104* after antibiotic treatment for two hours, which also indicates no damage on the bacterial cell wall during antibiotic treatment. The loss of the SERS signal from *Salmonella Typhi* bacteria during Augmentin antibiotic treatment for the *Salmonella Typhi* bacteria is due to the decrease in the number of “hot spots” on the bacteria surface due to cell wall damage. Since the SERS intensity is only an indication of the number of bacteria experiencing EF, during antibiotic treatment, the number of unaffected bacteria on the hotspots will decrease with time and as a result, the SERS intensity from the *Salmonella Typhi* bacteria decreases.

To better understand cell wall and membrane damage during antibiotic treatment, we performed a bacterial ATP leakage experiment. Experimental details have been reported before.^29,30^ The ATP leakage experiment data as reported in Fig. 3J clearly shows high leakage of cellular ATP during Augmentin antibiotic treatment for the *Salmonella Typhi* bacteria. On the other hand, as reported in Fig. 3J, experimental data also indicate minimum leakage of cellular ATP during Augmentin antibiotic treatment for the *Salmonella DT104* bacteria. All the above data clearly shows that cell wall and membrane damage during antibiotic treatment is high for *Salmonella Typhi* and it is minimal for *Salmonella DT104*.

Cell viability data as measured by the bacterial viability assay kit and as reported in Fig. 3H also indicate that multidrug resistant *Salmonella DT104* bacteria remain alive during Augmentin antibiotic treatment for three hours. On the other hand, more than 90% of the *Salmonella Typhi* bacteria died during Augmentin antibiotic treatment for three hours. All the above reported experimental data clearly shows that the 2D–0D heterostructure based SERS platform has the capability for separation of the *Salmonella DT104* drug resistant strain and the *Salmonella Typhi* normal strain *via* the antibiotic susceptibility test. Fig. 3I shows how the SERS intensity at 1140 cm^−1^ varies as the number of live *Salmonella Typhi* bacteria decreases during treatment with the Augmentin antibiotic. Since among all the observed Raman bands from the bacteria, the Raman mode at 1140 cm^−1^ is one of the strongest band for *Salmonella Typhi* and it is also a common band for both the bacteria we studied here, we used this band for plotting against the number of live bacteria. We measured the time dependent SERS intensity during treatment and reported the ratio of SERS intensity at 1140 cm^−1^ before and after treatment. Our data indicate that SERS intensity at 1140 cm^−1^ decreases abruptly as the number of live *Salmonella Typhi* bacteria decreases during treatment with the Augmentin antibiotic. From the experimental data we determined that even at ∼10^2^ CFU mL^−1^ concentration, a distinguishable SERS spectrum change can be obtained during antibiotic treatment.

## Conclusions

In the current manuscript, we reported the design of a novel heterostructure material using 2D-WS_2_ and 0D-GNPs, which can be used to determine the *antimicrobial susceptibility via* surface enhanced Raman spectroscopy (SERS). Experimental data indicate that in the heterostructure EM and CM enhancement factors have been combined and a very high SERS sensitivity of ∼10^−10^ to 10^−11^ M was observed, which is several orders of magnitude higher than 2D-TMD (∼10^−3^) and 0D-GNPs (∼10^−6^ to 10^−7^) individually. Reported FDTD simulation data show that due to the formation of “hot spots” by 0D-GNPs in the heterostructure material, the SERS enhancement can be four orders of magnitude higher than that of an individual nanoparticle.

Experimental data demonstrate that 0D-2D heterostructure based SERS has the capability for the rapid determination of the antimicrobial susceptibility of multidrug resistant *Salmonella DT104* and the normal strain *Salmonella Typhi*. Reported data show that since cell wall and membrane damage during antibiotic treatment is high for *Salmonella Typhi*, Raman bands from *Salmonella Typhi* bacteria decreased significantly during antibacterial treatment. Experimental data also indicate that since cell wall and membrane damage during antibiotic treatment are minimal for *Salmonella DT104*, the Raman bands remain unaltered during antibacterial treatment. Reported data demonstrate that the 2D-0D heterostructure-based SERS platform has the capability for the rapid identification of the *Salmonella DT104* drug resistant strain and separation from the *Salmonella Typhi* normal strain *via* an antibiotic susceptibility test, even at a concentration of 100 CFU mL^−1^.

## Conflicts of interest

There are no conflicts to declare.

## Supplementary Material
